# Comparative Genomics of the Ectomycorrhizal Sister Species *Rhizopogon vinicolor* and *Rhizopogon vesiculosus* (Basidiomycota: Boletales) Reveals a Divergence of the Mating Type *B* Locus

**DOI:** 10.1534/g3.117.039396

**Published:** 2017-04-20

**Authors:** Alija Bajro Mujic, Alan Kuo, Andrew Tritt, Anna Lipzen, Cindy Chen, Jenifer Johnson, Aditi Sharma, Kerrie Barry, Igor V. Grigoriev, Joseph W. Spatafora

**Affiliations:** * Department of Botany and Plant Pathology, Oregon State University, Corvallis, Oregon 97331; † Joint Genome Institute, United States Department of Energy, Walnut Creek, California 95458

**Keywords:** fungal mating pheromone, isoprenylcysteine carboxyl methyltransferase, Boletales, ectomycorrhizae, truffle, Genetics of Sex

## Abstract

Divergence of breeding system plays an important role in fungal speciation. Ectomycorrhizal fungi, however, pose a challenge for the study of reproductive biology because most cannot be mated under laboratory conditions. To overcome this barrier, we sequenced the draft genomes of the ectomycorrhizal sister species *Rhizopogon vinicolor* Smith and Zeller and *R. vesiculosus* Smith and Zeller (Basidiomycota, Boletales)—the first genomes available for Basidiomycota truffles—and characterized gene content and organization surrounding their mating type loci. Both species possess a pair of homeodomain transcription factor homologs at the mating type *A*-locus as well as pheromone receptor and pheromone precursor homologs at the mating type *B*-locus. Comparison of *Rhizopogon* genomes with genomes from Boletales, Agaricales, and Polyporales revealed synteny of the *A*-locus region within Boletales, but several genomic rearrangements across orders. Our findings suggest correlation between gene content at the *B*-locus region and breeding system in Boletales with tetrapolar species possessing more diverse gene content than bipolar species. *Rhizopogon vinicolor* possesses a greater number of *B*-locus pheromone receptor and precursor genes than *R. vesiculosus*, as well as a pair of isoprenyl cysteine methyltransferase genes flanking the *B*-locus compared to a single copy in *R. vesiculosus*. Examination of dikaryotic single nucleotide polymorphisms within genomes revealed greater heterozygosity in *R. vinicolor*, consistent with increased rates of outcrossing. Both species possess the components of a heterothallic breeding system with *R. vinicolor* possessing a *B*-locus region structure consistent with tetrapolar Boletales and *R. vesiculosus* possessing a *B*-locus region structure intermediate between bipolar and tetrapolar Boletales.

The mating event of fungi in Basidiomycota involves the anastomosis of monokaryons (one nucleus per cell) and the formation of a dikaryon (two nuclei per cell) (Heitman *et al.* 2013). Because the spores of most species of ectomycorrhizal (ECM) fungi cannot be germinated and maintained as monokaryons in culture, they pose a challenging system for the study of fungal reproductive biology. Some progress has been made with cultivable ECM Agaricomycetes, and those species that have been studied can generally be categorized into one of three breeding systems: secondary homothallic (pseudohomothallic—a form of selfing resulting from postmeiotic migration of compatible nuclei into spores), as in *Laccaria altaica* (Fries and Mueller 1984); heterothallic bipolar (50% chance of self compatibility), as in *Suillus luteus*, *Su. granulatus*, and *Rhizopogon roseolus* (Fries and Neumann 1990; Kawai *et al.* 2008); and heterothallic tetrapolar (25% chance of self-compatibility), as in *Hebeloma cylindrosporum* and *Laccaria bicolor* (Debaud and Gay 1987; Niculita-Hirzel *et al.* 2008). This range of breeding systems encompasses the most common systems known in fungi of Basidiomycota (Heitman *et al.* 2013), except for primary homothallic (completely self-compatible). There are currently no ECM fungal species demonstrated to possess a primary homothallic breeding system with the possible exception of *Sistotrema brinkmanii* (Ullrich and Raper 1975; Moncalvo *et al.* 2006).

The recent development of next generation sequencing technologies has allowed for novel approaches to the study of fungal genetics (Martin *et al.* 2011). To date, the only ECM Agaricomycetes to have its mating type (*MAT*) loci characterized with these techniques is the heterothallic tetrapolar mushroom *L. bicolor* (Niculita-Hirzel *et al.* 2008). In this study, we leverage these technologies to investigate the breeding systems of the ECM Agaricomycetes *Rhizopogon vinicolor* and *R. vesiculosus* (Basidiomycota, Boletales), which have been the focus of population genetics and ecology studies (Kretzer *et al.* 2003, 2005; Beiler *et al.* 2010, 2012; Dunham *et al.* 2013; Mujic *et al.* 2016). Species of *Rhizopogon* produce hypogeous fruiting bodies, also called false truffles, which achieve spore dispersal when they are excavated and consumed by mammals (Maser *et al.* 1978; Maser and Maser 1988). It likely that most *Rhizopogon* species possess a heterothallic breeding system because *R. roseolus*—the only *Rhizopogon* species with a known breeding system—is known to be heterothallic bipolar (Kawai *et al.* 2008). *R. vinicolor* and *R. vesiculosus* are sister species that share a sympatric distribution in the Pacific Northwest of North America, and grow in association with only a single ECM host species: *Pseudotsuga menziesii* (Douglas fir) (Molina and Trappe 1994). When co-occurring in a stand of *Pseudotsuga*, they can often be detected in near equal frequencies as both ECM root tips and fruiting bodies (Kretzer *et al.* 2003, 2005; Dunham *et al.* 2013). Despite similarities, these species display different life histories and population structures. *R. vesiculosus* produces larger genets on average (Kretzer *et al.* 2003, 2005; Beiler *et al.* 2012; Dunham *et al.* 2013), producing more and larger sporocarps per genet than *R. vinicolor*, and shows patterns of inbreeding within a range of 120 meters (Dunham *et al.* 2013). Effects of localized inbreeding are observable at the landscape scale, with populations of *R. vesiculosus* showing increased levels of population differentiation over shorter distances than those of *R. vinicolor* (Kretzer *et al.* 2005).

Several hypotheses have been proposed to explain the patterns of population structure observed in *R. vinicolor* and *R. vesiculosus*. Differential rates of secondary homothallism, *i.e.*, the production of heterokaryotic binucleate basidiospores, is one possible explanation. However, both species produce binucleate spores at near equal and relatively low rates (1–2%) (Dunham *et al.* 2013), which are typical of outcrossing ECM Agaricomycetes (Horton 2006). It is possible that *R. vesiculosus* is more likely to mate with close relatives because of its larger genet size, and higher production of sporocarps per genet (Dunham *et al.* 2013), or that *R. vinicolor* is under selective pressure from interspecies competition with *R. vesiculosus* to outcross more readily (Mujic *et al.* 2016). However, without further knowledge of the breeding system and the number of *MAT* alleles operating in these two fungi, we cannot conclusively determine the source of observed population structure in their natural populations.

Here, we report the first published genome sequences of truffle-forming Basidiomycota, *R. vinicolor* and *R. vesiculosus*, with particular emphasis upon the gene content and synteny of the regions surrounding their *MAT* loci. Sequencing and analysis of these genomes was performed with the intention of testing two hypotheses: (1) the differential population structure of *R. vinicolor* and *R. vesiculosus* is correlated with differences in the genetic content and organization of *MAT* loci. (2) Decreased heterozygosity resulting from localized inbreeding in *R. vesiculosus* is detectable as a reduced rate of nonsynonymous SNP mutation in dikaryotic genome assemblies. In order to test these hypotheses, we have produced detailed maps of gene content and organization surrounding the *A*-locus homeodomain transcription factor (*HD*) genes and *B*-locus lipopeptide pheromone precursor and pheromone receptor genes (we refer to precursor and receptor genes collectively as *P/R*) typical of the *MAT* loci in the Agaricomycetes. We have also developed a novel analytical pipeline to characterize rates of SNP mutation within heterokaryotic genome assemblies. This pipeline utilizes inputs of predicted protein coding genes and SNP mutations determined from previously existing software to analyze the expected effect of SNPs upon the amino acid sequences of proteins. Here, we demonstrate this method as an effective means of characterizing the heterozygosity of genome assemblies generated from heterokaryotic samples.

## Materials and Methods

### Culture conditions, tissue harvest, and nucleic acid extraction

Tissue cultures of *R. vinicolor* and *R. vesiculosus* were derived from fresh sporocarps collected during the summer of 2011 from a field site located on Mary’s Peak in the Oregon Coast Range. Coordinates and ecological properties of this site are detailed in Dunham *et al.* (2013). Each sporocarp was cleaned of adhering debris using a damp cloth, and divided into two hemispheres using a flame sterilized scalpel. Small sections (1 mm^3^) of clean dikaryotic tissue from the internal basidiospore bearing region (gleba) were then transferred using sterile technique to 60 mm Petri dishes of modified Melin-Norkrans media (MMN) (Kennedy *et al.* 2011), and incubated at 25° until growth was observed. Successful tissue cultures were screened for healthy growth, and a single culture of both *R. vinicolor* and *R. vesiculosus* was selected for genome sequencing. The sporocarps used to derive these cultures were accessioned into the fungal herbarium collections of Oregon State University (OSU) under the accession numbers OSC # 147973 (*R. vinicolor*, AM-OR11-026) and OSC # 148003 (*R. vesiculosus*, AM-OR11-056). Tissue was grown for DNA extraction using a single growth medium for each species, and tissue was grown for RNA extraction using four separate growth media for each species to maximize the diversity of RNA transcripts harvested. Full details of culture conditions, growth media, and nucleic acid extraction can be found in Supplemental Material, File S1. DNA extractions were quantified using a Qubit flourometer (Life Technologies, Grand Island, NY), and multiple extracts of each isolate were combined and precipitated with 95% ethanol to produce a single concentrated DNA solution for genome sequencing. RNA extractions were quantified using a Qubit flourometer, and quality was accessed using a NanoDrop spectrophotometer (Thermo Scientific, Wilmington, DE). RNA extractions from each of the four growth conditions were combined in equal proportions for each isolate to create a single pooled RNA extraction for transcriptome sequencing.

### Genome sequencing and assembly

Full details of genomic and transcriptomic library construction can be found in File S1. Illumina library construction, sequencing, and annotation of the *R. vinicolor* genome and transcriptome were conducted at the United States Department of Energy Joint Genome Institute (DOE-JGI) (Walnut Creek, CA). The *R. vinicolor* genome and transcriptome libraries were multiplexed with other sequencing projects into pools of two libraries, and each pooled set was sequenced on a single lane of an Illumina HiSeq2000 sequencer flowcell at the DOE-JGI using a TruSeq SBS sequencing kits, v3, following a 2 × 150 indexed run recipe. Genome reads were QC filtered for artifact/process contamination and subsequently assembled together with Velvet (Zerbino and Birney 2008). The resulting assembly was used to simulate a long mate-pair library with insert 3000 ± 300 bp, which was then assembled together with the original Illumina library with AllPathsLG (Gnerre *et al.* 2011). RNA-seq data were used as input for *de novo* assembly of RNA contigs. Reads were assembled into consensus sequences using Rnnotator (Martin *et al.* 2010). Both inputs were used to annotate genomes with the DOE-JGI Annotation pipeline, and release data in the DOE-JGI fungal genome portal MycoCosm (http://jgi.doe.gov/fungi; Grigoriev *et al.* 2014).

Illumina library construction, sequencing, assembly, and annotation of the *R. vesiculosus* genome and transcriptome were performed at OSU. Illumina sequencing of *R. vesiculosus* libraries was performed on an Illumina HiSeq 2000 at the OSU Central Services Laboratory (CSL) in the Center for Genome Research and Biocomputing (CGRB). The genomic DNA library was sequenced on a full flow cell lane using a 2 × 100 bp cycle, and Illumina version 2 chemistry. The RNAseq library was sequenced on 1/8 of a flowcell lane using a 1 × 50 bp cycle and Illumina version 3 chemistry. Raw Illumina reads were trimmed and quality filtered using the fastx toolkit (http://hannonlab.cshl.edu/fastx_toolkit/) and custom Perl scripts. *De novo* genome assembly of DNA reads was performed using VELVET (Zerbino and Birney 2008), and *de novo* transcriptome assembly was performed using TRINITY (Grabherr *et al.* 2011). Gene annotation of *R. vesiculosus* was performed using the MAKER pipeline (Cantarel *et al.* 2008) with *R. vesiculosus* transcriptome data and reference EST and protein homology data downloaded from the DOE-JGI MycoCosm portal for four closely related members of order Boletales (*R. vinicolor*, *Boletus edulis*, *Suillus brevipes*, and *Su. luteus*).

### Synteny analyses

To determine the organization of genomic regions containing the *A*-locus *HD* and *B*-locus *P/R* genes of *R. vinicolor* and *R. vesiculosus*, we used a multistep reference-guided approach. First, we identified contigs in the genome assemblies of *R. vinicolor* and *R. vesiculosus* that contained genes with predicted *MAT* function. Genes of known *MAT* function in other fungi were used as queries in BLASTP searches of custom BLAST databases of *R. vinicolor* and *R. vesiculosus* predicted gene models. The *A*-locus *HD* genes of Agaricomycetes are consistently found in close proximity to the gene encoding the mitochondrial intermediate peptidase (*MIP*) protein (James *et al.* 2013). The reference sequences used as queries in *A*-locus protein BLAST searches were the *HD* genes of *R. roseolus* (GenBank accession #s: BAL45602, BAL45603) and *MIP* gene of *Suillus pictus* (GenBank accession #: AY179596). *B*-locus *P/R* genes of Agaricomycetes are typically found in close association with one another as functional “cassettes” containing a pheromone receptor and one or more pheromone precursors (Casselton and Olesnicky 1998; Brown and Casselton 2001). *B*-locus protein BLAST searches used pheromone receptor genes of *Coprinopsis cinerea* (GenBank accession #: AAO17256) and *Serpula lacrymans* (GenBank accession #: EGO31061), which are known to function in mating recognition (Stajich *et al.* 2010; Skrede *et al.* 2013) as well as a pheromone precursor gene of *C. cinerea* (*C. cinerea phb3.2*, Stajich *et al.* 2010).

We characterized the gene content and arrangement of *R. vinicolor* and *R. vesiculosus* assembly contigs that contained the *A*-locus *HD* genes with the *MIP* gene flanking their position as well as contigs containing *P/R* gene cassettes. All of the genes identified on these contigs were used as queries in BLAST searches to identify homologous genomic regions in reference fungal genomes available on the DOE-JGI MycoCosm. Homologous proteins were identified in reference genomes as the top BLAST hit to *Rhizopogon* proteins with *e*-values ≥ 1*e*^−20^. Sequence composition of pheromone precursor genes is highly divergent within and between taxa, and, while the transmembrane domains of pheromone receptor genes are relatively conserved in Agaricomycetes, not all fungal species contain homologs of the same pheromone receptor genes (James *et al.* 2006, 2013; Kües 2015). Thus, we used the VISTA synteny browser available through the DOE-JGI MycoCosm to perform visual inspection of gene annotations in the putative *MAT* regions identified by BLAST searches, and performed additional BLAST searches of candidate protein sequences from the *B*-locus regions of *R. vesiculosus*. We did not readily identify pheromone precursor genes in *Rhizopogon* genomes or in reference genomes using BLAST searches. DOE-JGI genome annotation included automatic PFAM domain searches that identified most fungal mating pheromone domains, but nearly all pheromone precursor genes were absent from DOE-JGI predicted gene models. To resolve pheromone precursor gene boundaries, we designed a custom Perl script (pheromone_seeker.pl, File S2) that uses regular expression pattern matching to search for open reading frames containing the terminal –CAAX prenylation motifs (C = Cysteine residue, A = any aliphatic residue, X = any amino acid residue) that are typical of fungal *B*-locus pheromone precursor genes (Caldwell *et al.* 1995; Casselton and Olsenicky 1998). The gene content of *MAT* gene containing regions identified in reference genomes were visually inspected to confirm conserved gene synteny with the *A* and *B MAT* regions identified in *Rhizopogon* genomes.

Published reference genomes were chosen from both bipolar and tetrapolar species and covered as much taxonomic breadth within Agaricomycetes as possible while still retaining enough genomic synteny to allow for alignment. For the *A*-locus region, we chose reference genomes from *Su. luteus* (Boletales:Suillaceae, DOE-JGI taxon ID: Suilu1, Kohler *et al.* 2015), *S. lacrymans* (Boletales:Serpulaceae, DOE-JGI taxon ID: SerlaS7_9_2, Eastwood *et al.* 2011), *L. bicolor* (Agaricales:Hydnangiaceae, DOE-JGI taxon ID: Lacbi2, Martin *et al.* 2008; Niculita-Hirzel *et al.* 2008), *C. cinerea* (Agaricales:Psathyrellaceae, DOE-JGI taxon ID: Copci1, Stajich *et al.* 2010), and *Phanerochaete chrysosporium* (Polyporales:Phanerochaetaceae, DOE-JGI taxon ID: Phchr2, Ohm *et al.* 2014; Niculita-Hirzel *et al.* 2008) that are available for download and review in the DOE-JGI MycoCosm. Reference genomes for the *B*-locus region were the same as those chosen for the *A*-locus, with the exclusion of *P. chrysosporium* and the addition of *Paxillus involutus* (Boletales:Paxillaceae, DOE-JGI taxon ID: Paxin1, Kohler *et al.* 2015), *Su. brevipes* (Boletales:Suillaceae, DOE-JGI taxon ID: Suibr1, Branco *et al.* 2015), *Su. granulatus* (Boletales:Suillaceae, DOE-JGI taxon ID: Suigr1, unpublished and used by permission of author), *Rhizopogon salebrosus* (Boletales:Rhizopogonaceae, DOE-JGI taxon ID: Rhisa1, unpublished and used by permission of author), *Pisolithus tinctorius* (Boletales:Sclerodermataceae, DOE-JGI taxon ID: Pisti1, Kohler *et al.* 2015), *Pisolithus microcarpus* (Boletales:Sclerodermataceae, DOE-JGI taxon ID: Pismi1, Kohler *et al.* 2015), and *Coniophora puteana* (Boletales:Coniophoraceae, DOE-JGI taxon ID: Conpu1, Floudas *et al.* 2012).

For annotation of the region surrounding the *A*-locus, a reference region of ∼400 kbp centered on the *HD* genes was chosen from the genome assembly of *S. lacrymans*. All predicted proteins falling within this region were identified and used as BLAST queries against the *Rhizopogon* genomes and reference genomes. Mapping of gene coordinates and compilation of protein sequences was performed manually, or with the use of custom Perl scripts (protein_coordinates.pl, protein_select_by_contig.pl, select_by_contig_region.pl, File S3, File S4, and File S5). Reference based annotation of the region surrounding the *B*-locus in *Rhizopogon* genomes was performed upon a region of ∼80 kbp centered around reference *P/R* genes in *S. lacrymans*. To enhance the clarity of Figure 3 *Su. granulatus* and *P. tinctorius* genomes were omitted from B-locus synteny analyses because *Su. granulatus* was nearly identical to selected genome*s* of *Suillus*, and *P. tintoriu*s was divergent in gene content in regions surrounding *P/R* genes. *Rhizopogon* genomes sequenced in this study and some of the reference genomes were fragmented into several contigs in the areas surrounding the *MAT A* and *B* loci. We conducted genomic alignments of putative *MAT A* and *B* locus regions using MAUVE (Darling *et al.* 2004) to facilitate the reconstruction of fragmented genomes, and final synteny maps were visualized using CHROMOMAPPER (Niculita-Hirzel and Hirzel 2008). The nucleotide sequences of *MAT* genes identified from *R. vinicolor* and *R. vesiculosus* genomes were extracted from contigs using custom Perl scripts (protein_coordinates.pl, select_by_contig_region.pl, File S3 and File S4). These sequences were used in additional BLAST searches of the *R. vinicolor* and *R. vesiculosus* genomes to locate additional *MAT* gene alleles that may have assembled on contigs separate from the main *MAT* loci.

### Phylogenetic analyses

To confirm the homology of pheromone receptor homologs, we conducted phylogenetic analysis of all putative *B*-locus pheromone receptor genes identified in this study along with several reference sequences of pheromone receptor genes known to function in mating recognition in Agaricomycetes. Alignment of raw amino acid sequence was performed using MUSCLE with default settings (Edgar 2004), and the variable C terminal regions of these proteins were trimmed from alignments. Phylogenetic analysis was conducted using the maximum likelihood algorithm implemented in RAxML 7.2.6 with the PROTGAMMAGTR model of evolution, and 1000 bootstrap replicates (Stamatakis 2006). In the synteny analysis of the region surrounding the *B*-locus, we discovered several homologs of genes encoding putative isoprenyl cysteine methyltransferase (*ICMT*) proteins—a family of proteins that function in post-translational modification of pheromone precursor genes (Caldwell *et al.* 1995). The copy number and placement of these *ICMT* genes within Boletales genomes was correlated with breeding system, but orthology of these genes was unclear. To clarify this relationship a phylogenetic analysis was conducted upon an *ICMT* gene homolog amino acid alignment using the methods outlined above.

### Heterozygosity

The genomes of *R. vinicolor* and *R. vesiculosus* were sequenced using DNA extracted from dikaryotic tissue, and heterozygosity in the form of indels and single nucleotide polymorphisms (SNP) were visible in the assembly pile-up file generated by VELVET. SNPs were identified by creating alignments of trimmed and quality filtered Illumina reads to the consensus sequence of the *de novo* assemblies using BOWTIE2 (Langmead and Salzberg 2012). Pileup files were generated from BOWTIE2 alignments using SAMtools (Li *et al.* 2009), and final SNP calls were performed using VARSCAN2 (Koboldt *et al.* 2012). SNPs were predicted by VARSCAN2 under default settings, with the exception of a threshold *P*-value cutoff of 1*e*^−4^. The rate of synonymous *vs.* nonsynonymous mutations in each genome was determined using a custom Perl script (SNP_Density_calc.pl, File S6). Similar methods estimating heterozygosity from haplotypic SNPs in a diploid assembly have been applied to *Candida albicans* (Jones *et al.* 2004), human (Venter *et al.* 2001), and *Anopheles* (Holt *et al.* 2002) genome assemblies. Our script streamlines this analysis by utilizing output from existing software packages, and quantifies genome-scale heterozygosity by characterizing SNP distribution and the rate of nonsynonymous mutation between haplotypes of a single fungal individual. This script is applicable to any polyploid assembly for which gene models are available in AUGUSTUS (Stanke *et al.* 2008), MAKER, or DOE-JGI .gff3 file format.

### Data availability

The *Rhizopogon* tissue cultures sequenced in this study are accessioned in the Center for Forest Mycology Research (CFMR) culture collection (US Forest Service, Madison, WI) under the numbers AM-OR11-026 (*R. vinicolor*) and AM-OR11-056 (*R. vesiculosus*). The genome assemblies and gene annotations for the *R. vesiculosus* and *R. vinicolor* draft genomes are available through the National Center for Biotechnology Information (NCBI) GenBank under accession numbers LVVM00000000 (*R. vesiculosus*) and LYZI00000000 (*R. vinicolor*). Both genomes are available through the DOE-JGI Mycocosm portal (http://jgi.doe.gov/fungi). Amino acid alignment files and tree files are available at Treebase (http://treebase.org) under the study ID TB2:S19165. File S1 contains detailed methods for tissue culture conditions, nucleic acid extractions, and genome sequencing and assembly. All Perl scripts written as a part of this study are available in File S2, File S3, File S4, File S5, and File S6. File S7 contains coordinates of all *A*-locus and *B*-locus regions gene models identified from all genomes examined in this study. File S8 contains captions for all supplementary files. Table S1 contains coordinates predicted gene models, as well as BLAST hits to the nucleotide sequences of *B*-locus region gene models that are present together on small contigs unlinked to the primary *B*-locus contigs in *R. vinicolor* and *R. vesiculosus* genomes. The data presented in this supplementary table likely represent partially assembled alleles of *B*-locus region genes.

## Results and Discussion

### Genome assembly statistics

The assembled genomes of *R. vinicolor* and *R. vesiculosus* were 36.1 and 42.2 Mbp, respectively, with the *R. vinicolor* assembly possessing the higher average genome coverage, and a lower degree of fragmentation (Table 1). The assembly of *R. vesiculosus* was 6.1 Mbp larger than that of *R. vinicolor*, with much of this additional genome size accounted for by intergenic regions of the assembly. Both assemblies contained a high proportion of sequence data within predicted protein coding regions with 23.1 Mbp (63.9%) of the *R. vinicolor* genome and 20.6 Mbp (48.9%) of the *R. vesiculosus* genome within the boundaries of predicted exons or introns. Table 1 presents a comparison of genome assembly statistics. We quantified genome assembly completeness using the default benchmarking algorithm implemented in CEGMA (Parra *et al.* 2007). Results of CEGMA benchmarking were similar between the two assemblies, with *R. vinicolor* possessing 235 complete CEGs (94.76% completeness), and *R. vesiculosus* possessing 236 complete CEGs (95.16% completeness).

**Table 1:** Summary of assembly statistics for the *R. vinicolor* and *R. vesiculosus* genomes

	Species	
	*R. vinicolor*	*R. vesiculosus*
Minimum contig size retained (bp)	1000	1000
Assembly length (Mbp)	36.1	42.2
Genic regions of assembly (Mbp/% of assembly)	23.1/63.9%	20.7/48.9%
Intergenic regions of assembly (Mbp/% of assembly)	13/36.1%	21.6/51.1%
Repetitive elements in genic regions of assembly (Mbp/# of elements)	0.18/1147	0.28/1949
Repetitive elements in intergenic regions of assembly (Mbp/# of elements)	0.78/2106	3.16/12,683
Average read coverage depth	154×	51×
Largest scaffold/contig (Mbp)	0.56	0.45
Number of scaffolds	2310	—
Number of contigs	3018	3765
Scaffold/contig N50	218	361
Raw N50	43,737	30,999
Number of gene models	14,469	13,623

Repeatmasker (http://repeatmasker.org) was used as a component of the MAKER annotation pipeline to identify repetitive elements in *R. vesiculosus*, and found 14,632 elements totaling 3.44 Mbp in length (8.1% of genome length). Repetitive elements were identified in *R. vinicolor* using the DOE-JGI annotation pipeline and 3253 elements totaling 0.96 Mbp in length (2.7% of genome length) were found in *R. vinicolor*. The majority of repetitive elements in both species were contained within the intergenic areas of the assembly with 12,683 elements totaling 3.16 Mbp in length (14.8% of the intergenic region of assembly) in *R. vesiculosus* and 2106 elements totaling 0.78 Mbp in length (6% of the intergenic region of assembly) in *R. vinicolor*. A greater percent content of repetitive elements in the *R. vesiculosus* assembly explains, in part, the greater length of this assembly. The two assemblies differ in length primarily with respect to the length of the assembled intergenic regions (*R. vinicolor* 13 Mbp *vs.* *R. vesiculosus* 21.6 Mbp) with genic regions similar in length (*R. vinicolor* 23.1 Mbp *vs.* *R. vesiculosus* 20.6 Mbp).

### Heterozygosity within dikaryons

We utilized the presence of SNPs in assemblies as a means of measuring heterozygosity in each dikaryotic individual. A reduced rate of nonsynonymous SNP mutation in the *R. vesiculosus* assembly compared with the *R. vinicolor* assembly supports our hypothesis that a reduced rate of nonsynonymous mutations is correlated with decreased heterozygosity in *R. vesiculosus*. We conclude that characterization of SNPs in a dikaryotic genome assembly is an effective means of determining the overall heterozygosity of the individual sequenced. VARSCAN2 predicted a total of 439,805 SNPs in the *R. vesiculosus* genome, and 483,084 SNPs in the *R. vinicolor* genome. While the majority of SNPs were identified from intergenic regions for both species, *R. vinicolor* possessed a greater proportion of its SNPs in predicted genes than *R. vesiculosus*, with 83.2% of all SNPs within intergenic regions and 16.8% in genic regions. In comparison, *R. vesiculosus* had 89.9% of all SNPs in intergenic regions, and 10.1% in genic regions. The rate of nonsynonymous mutation was also greater in *R. vinicolor*, with 6.1% of all SNPs causing nonsynonymous mutations compared to 3.3% of all SNPs causing nonsynonymous mutations in *R. vesiculosus*. A comparison of SNP ratios for *R. vinicolor* and *R. vesiculosus* assemblies is presented in Table 2.

**Table 2:** Summary of SNP mutations identified between the haplotypes of dikaryotic *Rhizopogon* genome assemblies

	Species	
	*R. vinicolor*	*R. vesiculosus*
Total SNPs	483,084	439,805
Percent of genome in SNPs	1.30%	1.00%
SNPs in intergenic regions	402,084/83.2%	395,390/89.9%
SNPs in genic regions	81,000/16.8%	44,415/10.1%
SNPs in exons	54,064/11.2%	29,580/6.7%
Synonymous exonic SNPs	24,694/5.1%	15,058/3.4%
Nonsynonymous exonic SNPs	29,370/6.1%	14,522/3.3%

Percent value for SNPs in rows 3–7 denote the percent represented by the row of all SNP mutations identified within a genome.

It is expected that heterozygosity between dikaryotic nuclei (haplotypes) should be most evident in nonconserved intergenic regions, or in genes that are expected to have highly divergent alleles, such as the *MAT* loci. Genome assembly in such variable regions of dikaryotic genome assemblies is methodologically challenging, and divergent regions can be assembled as a single haplotype, a consensus sequence combining sequences of both haplotypes, broken into multiple contigs where the divergent haplotypes terminate the ends of two contigs, or fail to assemble altogether (Jones *et al.* 2004). The genome assembly of *R. vesiculosus* is 6.1 Mbp larger than that of *R. vinicolor*, and, although the number of predicted gene models is similar between these assemblies, the percentage of the *R. vinicolor* assembly present in genic regions (63.9%) is greater than in the *R. vesiculosus* assembly (48.9%). This suggests that we have assembled a greater proportion of the intergenic genome regions of *R. vesiculosus* than we have in *R. vinicolor*. The reduced assembly of *R. vinicolor* in intergenic regions would be consistent with a higher rate of heterozygosity within this individual, which may have caused assembly failure of intergenic regions. A more complete assembly of noncoding regions in *R. vesiculosus* is likely due to reduced overall heterozygosity, and is not surprising, given that the individual sequenced in this study was drawn from a population that shows signs of inbreeding (Dunham *et al.* 2013).

### Gene content and synteny of the A-locus

**Figure 1:**
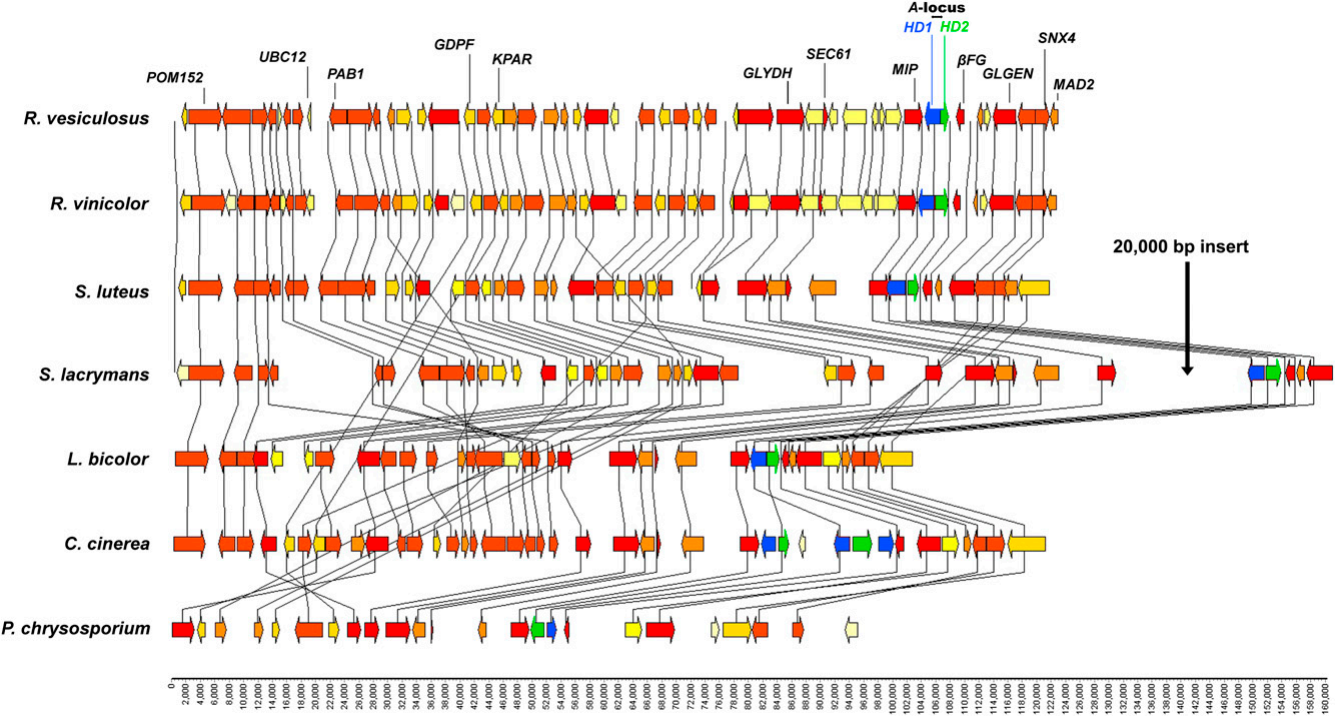
Synteny map for the highly conserved ∼130 kbp region surrounding the genes for the *A*-locus *HD* proteins of both *R*. *vinicolor* and *R. vesiculosus* and reference genomes. Each gene is represented by an arrow showing the direction of transcription. Gene color follows a heat map from yellow (gene represented in one genome) to red (gene represented in all genomes). *HD1* genes are highlighted in blue and *HD2* genes are highlighted in green. Gene acronyms are in reference to James *et al.* (2004b). The bottom scale is in nucleotide basepairs. For clarity, only genes present in *Rhizopogon* and *Suillus* genomes are shown in other genomes.

**Figure 2:**
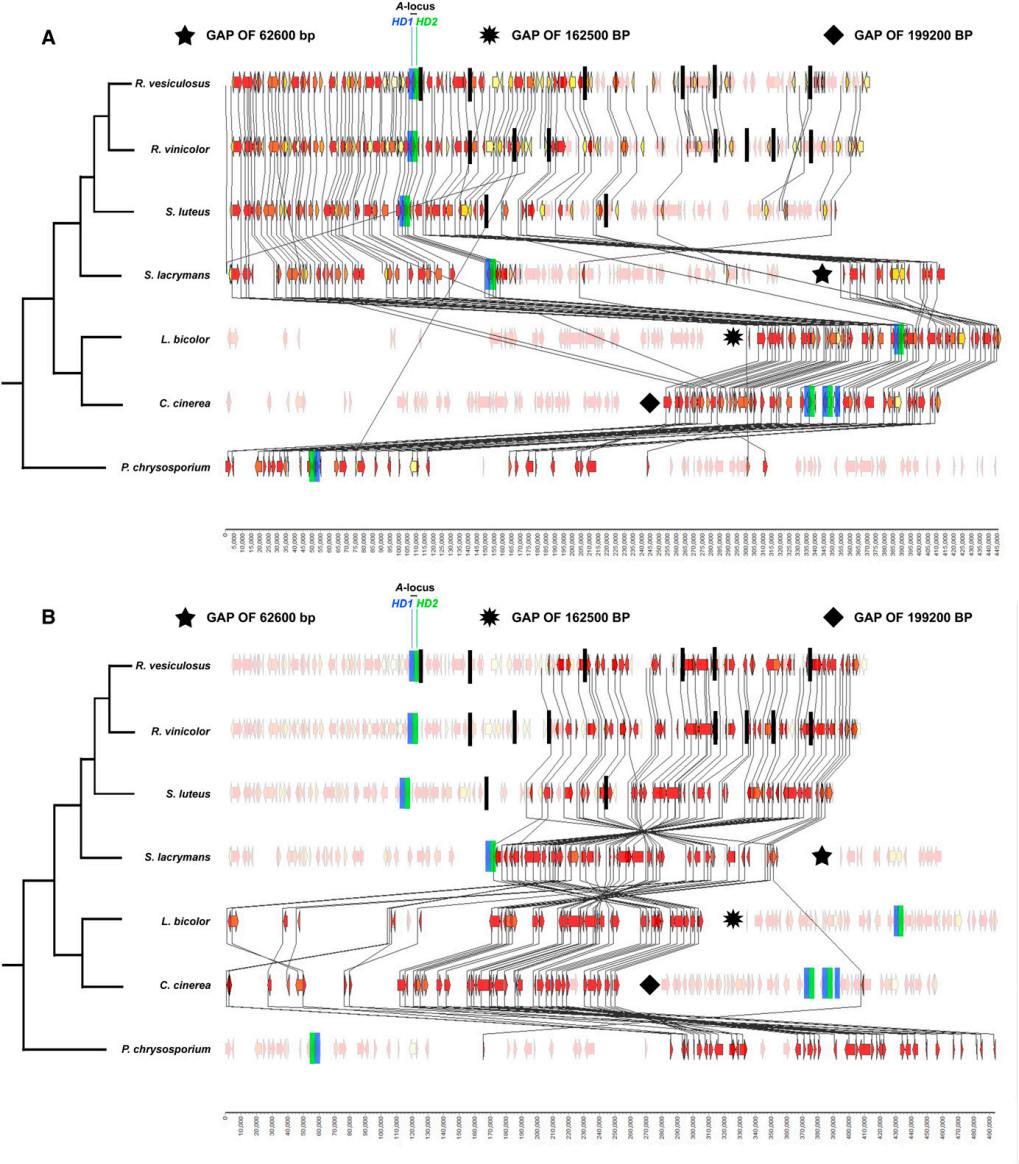
Synteny map for the region surrounding the genes for the *A*-locus *HD* proteins of *R. vinicolor* and *R. vesiculosus* and reference genomes. Each gene is represented by an arrow showing the direction of transcription. Gene color follows a heat map from yellow (gene represented in one genome) to red (gene represented in all genomes). Contig breaks for *R. vinicolor*, *R. vesiculosus*, and *Su. luteus* are shown as vertical black bars. Contigs were assembled into the scaffolds shown here based upon synteny with the genome of the closest relative that lacked a break at the same location. The genes encoding *HD1* proteins are marked in blue, and the genes encoding *HD2* proteins are marked in green. The phylogenetic relationships shown at the left of the figure are adapted from Hibbett (2006). Note that the gaps depicted by the stars and diamond are not drawn to scale. (A) Highlighted synteny of the conserved region shown in Figure 1, as well as the area at the far 3′ end of the characterized region. (B) Highlighted synteny of the region 3′ of the genes encoding *HD* proteins in Boletales and Polyporales genomes that is translocated to the 5′ side of the genes encoding *HD* proteins in Agaricales genomes. Note that this region is inverted in *S. lacrymans* relative to other genomes.

All of the genomes examined possessed a single, divergently transcribed, pair of *HD* genes, except for *C. cinerea*, which possessed two pairs of divergently transcribed *HD* genes, and one additional *HD1* gene in close proximity (Figure 1, Stajich *et al.* 2010). The *A*-locus regions of *R. vinicolor*, *R. vesiculosus*, and *Su. luteus* were assembled as multiple contigs, and were arranged into putative scaffolds here based upon MAUVE alignments with contiguous assemblies of homologous regions in other genomes (Figure 2; a complete listing of all genes identified from *A*-locus regions can be found in File S7). No additional alleles of *HD* genes were found assembled on any contigs separate from the primary *MAT A* region contigs. However, the 5′ and 3′ regions of the *R. vesiculosus HD2* gene are broken into two partial gene models at the ends of two contigs (Figure 2 and File S7). It is likely that these partial gene models represent partial assemblies of *HD2* alleles, and that the assembly of a complete allele failed due to highly divergent sequences between alleles at the region of the contig break.

Our analyses showed a high level of gene conservation between the *Rhizopogon* genomes and reference genomes for a 400 kbp region containing the genes encoding *HD* proteins. The genomic region beginning at ∼100 kbp 5′ and extending to 30 kbp 3′ of the genes encoding *HD* proteins in both *Rhizopogon* species shows the highest level of gene conservation and synteny with homologous regions of all reference genomes (Figure 1), as observed in other Agaricomycetes (James *et al.* 2004b, 2006, 2013; Niculita-Hirzel *et al.* 2008). The genes for the *HD* proteins in both *Rhizopogon* genomes are flanked 5′ by the gene encoding the *MIP* protein, and 3′ by the gene encoding the beta-flanking (*beta-fg*) protein; a gene organization that is consistent for nearly all characterized Agaricomycetes (James *et al.* 2004a, 2013; James 2007; Kües *et al.* 2011) with limited exceptions (Skrede *et al.* 2013; Ohm *et al.* 2010; Van Peer *et al.* 2011; Kües *et al.* 2015).

The genomic region beginning 35 kbp 3′ of the genes for the *HD* proteins in both *Rhizopogon* species contains several blocks of genes that are conserved in reference genomes, but possess a translocated and/or inverse orientation relative to *Rhizopogon* species. This block of translocated genes possesses conserved synteny within the gene cluster, and shares a common point of recombination ∼100 kbp 3′ of the genes for the *HD* proteins in *Rhizopogon* species (Figure 2). A greater degree of synteny is shared by *Rhizopogon* species, *Suillus* species, and *P. chrysosporium* in this region as compared to the Agaricales reference genomes *L. bicolor* and *C. cinerea*. In Agaricales reference genomes, a 120 kbp region beginning at this point is translocated ∼300 kb to the 5′ side of the genes encoding the *HD* proteins, with several inversions of gene blocks within this translocation. In addition to translocations in *C. cinerea* and *L. bicolor*, we also noted a major inversion in this neighboring region of the *A*-locus in *S. lacrymans* relative to other genomes examined. Inversions and translocations near the *A*-locus have previously been observed within order Agaricales for both *Flammulina velutipes* and *Schizophyllum commune* relative to an ancestral arrangement in *L. bicolor* and *C. cinerea* (Van Peer *et al.* 2011). Given our findings, and those of Van Peer *et al.* (2011), it seems likely that gene rearrangements are common in Agaricomycetes fungi in the regions adjacent to the *A*-locus. Our findings indicate that *L. bicolor* and *C. cinerea* possess a derived gene arrangement surrounding the *A*-locus relative to the Polyporales and Boletales species examined in this study. The evolutionary forces favoring gene rearrangements in the areas surrounding *A*-locus *HD* genes are unclear. However, strong balancing selection is known to function in the *HD* genes of *C. cinerea* (May *et al.* 1999), and it is possible that recombination near the *HD* genes may function to break linkage of surrounding regions with the *HD* genes.

### Gene content and synteny of the B-locus

**Figure 3:**
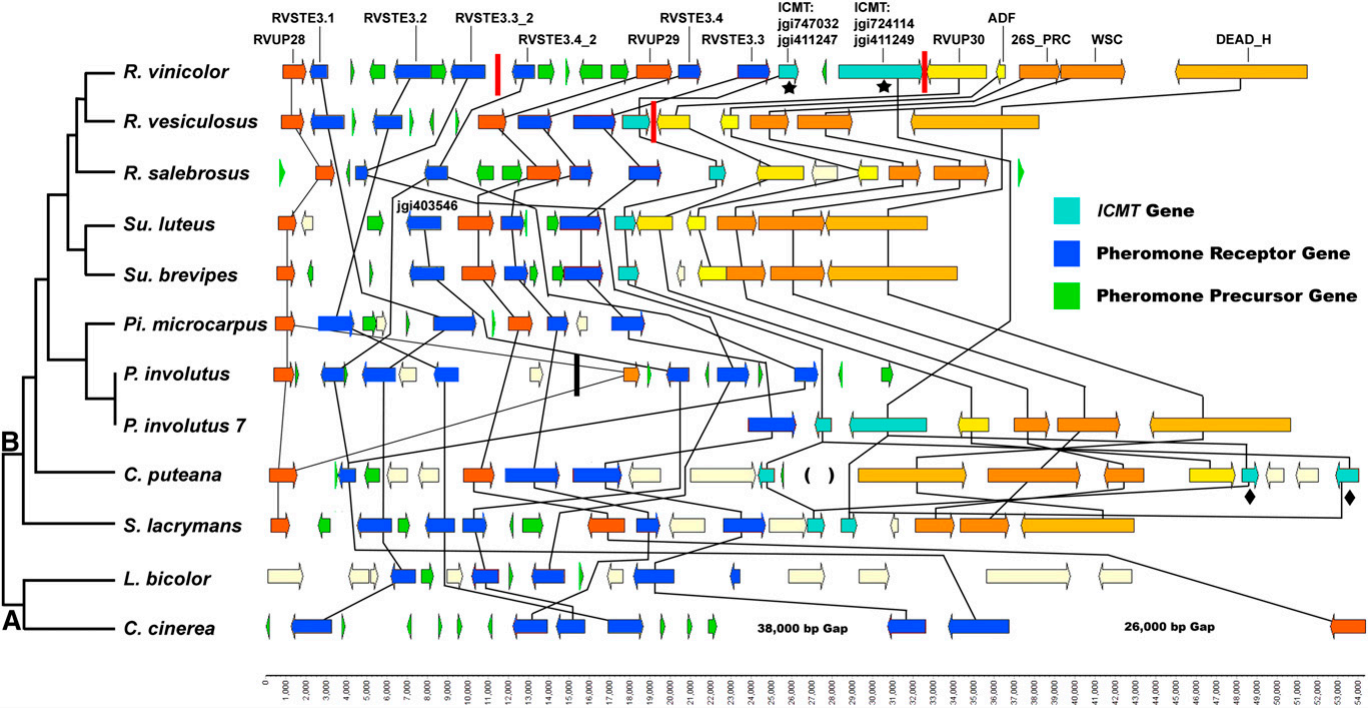
Synteny map for *B*-locus region surrounding the pheromone precursor and pheromone receptor genes identified in *R. vinicolor*, *R. vesiculosus*, *R. salebrosus*, *Su. luteus*, *Su. brevipes*, *Pisolithus microcarpus*, *P. involutus*, *C. puteana*, *S. lacrymans*, *L. bicolor*, and *C. cinerea*. Pheromone receptor genes are shown in blue, pheromone precursor genes in green, and isoprenyl cysteine methyltransferase (*ICMT*) genes in light blue. A vertical black bar marks the alignment of two *P. involutus* contigs that are of ambiguous alignment and shown here on a single row for clarity of presentation. “*Paxillus involutus* 7” represents an area containing *B*-locus region genes that has been transposed into a nonhomologous genomic region at the center of scaffold 7 in the *P. involutus* assembly. Red bars mark the location of breaks between *Rhizopogon* assembly contigs, which are assembled into scaffolds here based upon syntentic arrangement of contiguous regions in one *Rhizopogon* genome that spanned breaks in the other. Black stars mark two *R. vinicolor ICMT* genes that were both assembled as unique alleles at the terminal segments of two assembly contigs but are shown here as a single set of alleles for clarity. *C. puteana* is marked with parentheses at the location of a 130 kbp nonhomologous insert. It is also marked with black diamonds at *ICMT* genes that are found adjacent to the *B*-locus in other Boletales genomes examined but that are 3′ of the 130 kbp insert in *C. puteana*. The pheromone receptor gene at the 5′ side of *Su. luteus* is marked with its jgi protein ID and represents a receptor lineage, which is absent in *Rhizopogon* genomes. Note that the gaps depicted are not drawn to scale. The bottom scale is in nucleotide basepairs. The Cladogram depicting species relationships is adapted from phylogenies published by Kohler *et al.* (2015) and Binder and Hibbett (2006). Cladogram labels: (A) Agaricales, (B) Boletales.

**Figure 4:**
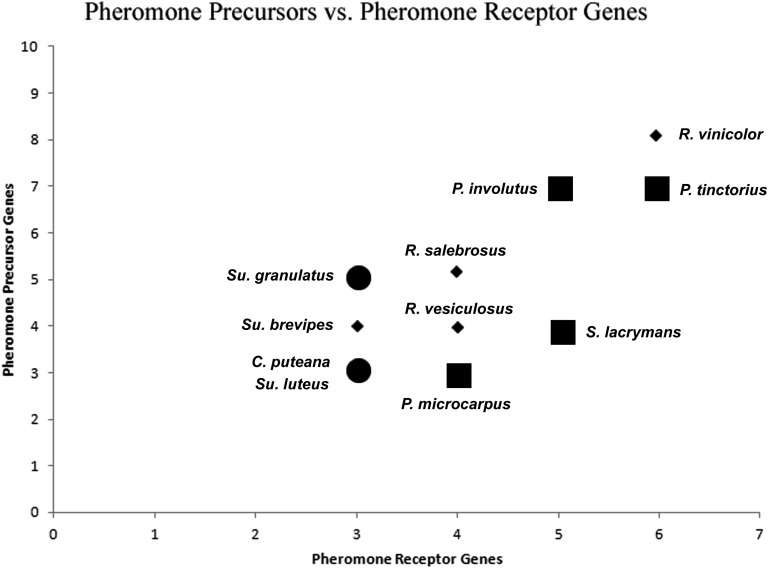
The number of *B*-locus mating-type pheromone precursor and pheromone receptor genes for all Boletales genomes examined. Species with a known tetrapolar breeding system are plotted by large squares, those with known bipolar systems are plotted by large circles, and those with unknown breeding systems are plotted by small diamonds. This figure includes data from all genomes of Boletales fungi with a known breeding system and an available genome assembly. Note that counts of genes here are from the primary *MAT B* region assembly contigs of each genome, and do not include putative pheromone receptor or pheromone precursor alleles assembled on contigs outside the *B*-locus region.

All genomes examined possess a *B*-locus region containing pheromone receptor genes in close proximity to pheromone precursor genes. Synteny between the *B*-locus regions is somewhat conserved at the ordinal level, with most species showing at least some synteny of *P/R* and nonmating type genes with other members of their order (Figure 3; a complete listing of the gene content from all *B*-locus regions examined can be found in File S7. The major exceptions are *P. involutus*, which shows low synteny with other Boletales genomes surrounding the *P/R* genes, *C. puteana*, which possesses a major inversion of the region 3′ of *P/R* genes, and *P. tinctorius* (File S7), which possesses two *B*-locus subloci that map to separate contigs. There was only limited synteny observed in the region of the *B*-locus between orders. This is consistent with previous studies of *B*-locus synteny, which found low levels of synteny between Agaricales and Polyporales species, as well as distantly related members of the Agaricales (Niculita-Hirzel *et al.* 2008; Van Peer *et al.* 2011). Tetrapolar species of order Boletales possessed a greater number of pheromone receptor genes than bipolar species, and each pheromone receptor gene tended to be located adjacent to a greater number of pheromone precursor genes than in bipolar species (Figure 3 and Figure 4). Of the species with uncharacterized breeding systems, *R. vinicolor* possessed a greater number of both pheromone receptor (6) and pheromone precursor (8) genes than *R. vesiculosus* (pheromone receptor 4, pheromone precursor 4) and *R. salebrosus* (pheromone receptor 4, pheromone precursor 5), while *Su. brevipes* shared very similar *B*-locus gene content and synteny with its close bipolar relatives *Su. luteus* and *Su. granulatus*. The breeding system and other characteristics of Boletales genomes examined at the *B*-locus region can be found in Table 3.

**Table 3:** Boletales genomes examined at the *B*-locus region

Species	Number of Contigs Spanning B-Locus Region	Breeding System	Ecological Habit	Karyotic State of Genomic DNA Source Tissue	Genome Reference
*Rhizopogon vesiculosus*	2	—	Ectomycorrhizal	Dikaryotic	This study
*Rhizopogon vinicolor*	3	—	Ectomycorrhizal	Dikaryotic	This study
*Rhizopogon salebrosus*	1	—	Ectomycorrhizal	Dikaryotic	Unpublished, used by permission of author
*Suillus brevipes*	2	—	Ectomycorrhizal	Dikaryotic	Branco *et al.* (2015)
*Suillus luteus*	2	Bipolar (Fries and Neumann 1990)	Ectomycorrhizal	Dikaryotic	Kohler *et al.* (2015)
*Suillus granulatus*	1	Bipolar (Fries and Neumann 1990)	Ectomycorrhizal	Dikaryotic	Unpublished, used by permission of author
*Coniophora puteana*	1	Bipolar (Ainsworth and Rayner 1990)	Saprobic	Monokaryotic	Floudas *et al.* (2012)
*Paxillus involutus*	3	Tetrapolar (Fries 1985)	Ectomycorrhizal	Dikaryotic	Kohler *et al.* (2015)
*Pisolithus tinctorius*	1	Tetrapolar (Kope and Fortin 1990)	Ectomycorrhizal	Dikaryotic	Kohler *et al.* (2015)
*Pisolithus microcarpus*	1	Tetrapolar (Sales *et al.* 2015)	Ectomycorrhizal	Dikaryotic	Kohler *et al.* (2015)
*Serpula lacrymans* S7.9	1	Tetrapolar (Harmsen 1960)	Saprobic	Monokaryotic	Eastwood *et al.* (2011)

**Figure 5:**
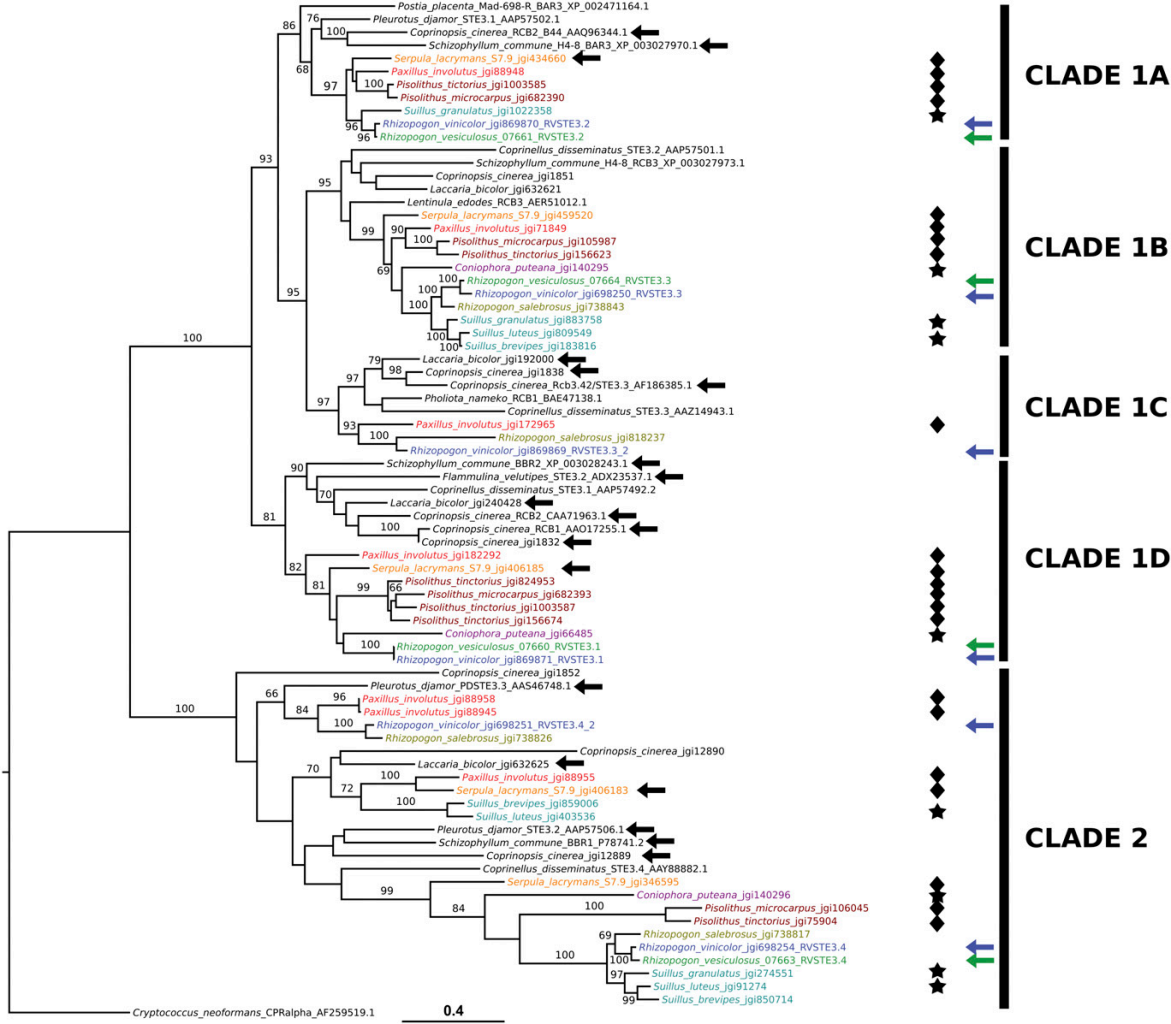
Maximum likelihood phylogram of pheromone receptor genes inferred using RAxML with 1000 bootstrap replicates and the PROTGAMMAGTR model of evolution. Bootstrap support values >60% are shown. Pheromone receptor genes derived from non-*Rhizopogon* Boletales genomes compared in Figure 4 are color coded by genus. *Rhizopogon* genes are color coded by species, and *R. vinicolor* and *R. vesiculosus* genes are further distinguished by dark blue and green arrows, respectively. Major groups of pheromone receptor genes as identified by James *et al.* (2004b), Riquelme *et al.* (2005), and Kües (2015) are here amended, and are denoted at the right margin. Black arrows mark genes of known mating function in *F. velutipes* (Van Peer *et al.* 2011), *L. bicolor* (Niculita-Hirzel *et al.* 2008), *S. lacrymans* (Skrede *et al.* 2013), *S. commune* (Ohm *et al.* 2010), and *C. cinerea* (O’Shea *et al.* 1998; Halsall *et al.* 2000; Riquelme *et al.* 2005; Stajich *et al.* 2010). Boletales taxa with known bipolar breeding systems are marked with stars, and those with known tetrapolar breeding systems are marked with diamonds.

The *B*-locus regions of *R. vesiculosus*, *R. vinicolor*, and *P. involutus* were assembled as multiple unlinked contigs, and were arranged into putative scaffolds by the same methods used for *A*-locus regions (Figure 3, File S7, and Table 3). It is possible the *P/R* genes identified at the ends of putatively adjacent *R. vinicolor* contigs (pheromone receptors *RvSTE3.3_2* and *RvSTE3.4_2*, and associated pheromone precursors) represent separately assembled alleles of the same *P/R* cassette rather than unique genes. Though *RvSTE3.3_2* and *RvSTE3.4_2* are inferred to be members of separate phylogenetic clades that both contain genes of known mating function (Figure 5), this is not evidence that they represent unique genes, because alleles of pheromone receptor genes from the same genomic location in both *S. commune* (Van Peer *et al.* 2011) and *C. cinerea* (Kües 2015) have been demonstrated to group in disparate phylogenetic clades. However, we believe that *RvSTE3.3_2* and *RvSTE3.4_2* represent unique genes due to syntenic arrangement of nonmating type genes in the region surrounding the *B*-locus of *R. vesiculosus* and *R. vinicolor*.

Many of the pheromone precursor genes identified in this study were not initially recognized by either the DOE-JGI or MAKER gene annotation pipelines, and ∼50% of the pheromone precursor genes discussed here were identified only by the use of our custom Perl pattern matching script, which searched for open reading frames with terminal -CAAX motifs. However, all the pheromone precursor genes identified in DOE-JGI and MAKER annotation pipelines were also identified by our Perl script. The pheromone precursor genes identified in *Rhizopogon* genomes contain both –CAAX and two residue “ER” motifs 10–15 residues 5′ of the –CAAX motif (Table 4) that support their role in mating recognition (Riquelme *et al.* 2005, Kües 2015). The –CAAX motif of fungal pheromone precursor genes is known to flag peptides for isoprenyl modification and the prenyl moiety incorporated into the modified protein is strongly influenced by the final “X” residue of the –CAAX motif (Caldwell *et al.* 1995). The pheromone precursor genes identified from *Rhizopogon* species are all terminated by residues that target them for farnesylation (Table 4, Caldwell *et al.* 1995), consistent with findings for other characterized fungal pheromones (Casselton and Olesnicky 1998; Brown and Casselton 2001; Michaelis and Barrowman 2012). We created custom BLAST databases of assembled *Rhizopogon* transcriptome data, and found that all putative pheromone precursor genes from *R. vinicolor* and *R. vesiculosus* were present in the population of transcripts, with the exception of *phb3.3* in *R. vesiculosus* and *phb2.1* in *R. vinicolor*.

**Table 4:** Predicted amino acid sequence of pheromone precursor genes identified in *Rhizopogon* genomes

*Rhizopogon vesiculosus* predicted pheromone precursors	
Rvphb2.1	MDSFDTFHTLEFPLDAPPDIGVEPESSSSVPLD**ED**SQYWRPGTF**CIIS**
Rvphb3.1	MDMFSSLSSISLEELDIVHTPSSSSSSYSLLAHSDGDSCPIPTEY**EH**SYSASSGWY**CIIA**
Rvphb3.2	MDAFDTILISELFQPDPPLSLDQGSCSSDDEPYLLVDA**DA**KWNYGGY**CVIS**
Rvphb3.3	MDEFVTLPSEPSSFAELSDSGDALQFEYSDALPVDD**DR**LSGYYGSF**CVII**
*Rhizopogon vinicolor* predicted pheromone precursors	
RVphb3.5	MDNFTSVSDIFFPTVSLEGDPSNSGDPNAVGDGEPVLVDA**EY**WTWGSSSQSGF**CVIV**
RVphb2.1	MDSFDTFHIPEFPLNAPPNPGVEPESSSSIPLD**ED**SQYWRPGTF**CIIS**
RVphb2.2	MDAFASIAELFSQSISLSEVTNIAPPDDHEMPVEY**ER**NPLSAGGF**CIIS**
RVphb3.3	MDEFITLPSDPSSFAELSHSGNASQFEYIAYSDTLPVDD**DR**LSGYYGSF**CVII**
RVphb2.3	MDNFTSVSDIFFPTVSLEGDPSNSGDPNAVGDGEPVLVDA**EY**WTWGSSSQSGF**CVIV**
RVphb3.3_2	MDQFITLPSEPSSFAELSHSGDASQFDVEYSDALPVDD**DR**LSGYYGSF**CVII**
RVphb3.1	MDIFSSLSSISLDELDIAHTPSSSSSYSRLAHSDDDACPIPTEY**EH**SYSASSGWY**CIIA**
RVphb3.2	MDAFDTILVSELFQPDPPLSLDQGSCSSDDEPYLLV**DA**DTKWNYGGY**CVIS**
RVphb3.4	MDIFSSLSSISLDELDIAHTPSSSSSYSRLAHSDDDACPIPTEY**EH**SYSASSGWY**CIIA**

The predicted N terminal couplet of mature pheromones (acid ER domain) and the C terminal –CAAX motif are presented in underlined boldface for each sequence.

All Boletales genomes, except *Pisolithus* species, possessed an *ICMT* homolog immediately 3′ of the *B*-locus. *ICMT* is a family of proteins responsible for activation of pheromone mating function by the addition of a methyl group to the C terminal cysteine of mature fungal mating pheromones (Caldwell *et al.* 1995). *ICMT* is represented by a single copy immediately 3′ of the *P/R* genes in *R. vesiculosus*, *R. salebrosus*, *Su. brevipes*, and the bipolar Boletales reference genomes, *Su. luteus*, *Su. granulatus*, and *C. puteana*. *C. puteana* possesses an additional two *ICMT* genes near the *B*-locus, but they are located 130 kbp downstream of the *B*-locus region in association with an inverted and translocated gene cluster typically found in close proximity to the *B*-locus in other Boletales genomes. *R. vinicolor* possesses two copies of *ICMT* in immediate proximity to one another immediately 3′ of the *P/R* genes as does the tetrapolar species *S. lacrymans*. The tetrapolar species *P. involutus* also possesses two copies of *ICMT i*n close proximity to the typical 3′ terminal pheromone receptor gene of the Boletales *B*-locus (the homolog of *R. vesiculosus RVSTE3.3*). This block of genes in *P. involutus*, along with other genes typically conserved within the *B*-locus region of other Boletales references, are imbedded at the center of a separate and much larger contig with no other observed *MAT* genes. The primary contigs containing other *P. involutus B*-locus genes are small, and contain only genes that are typically associated with the *B*-locus region in other Boletales references (Figure 3).

In *R. vinicolor*, the *B*-locus region is broken into separate contigs at the location of the *ICMT* pair, and each contig contains distinct alleles of *ICMT* genes (Figure 3 and File S7). This suggests that contig extension was terminated in this region during the assembly process, due to the presence of divergent alleles between haplotypes—an assembly artifact previously observed in the diploid genome assembly of the yeast *Candida albicans* (Jones *et al.* 2004). BLAST searches of the *R. vinicolor* genome using the nucleotide sequence of the *R. vesiculosus B*-locus region *ICMT* gene (Rves056.07665) as query identified two partial *ICMT* alleles on small contigs of the *R. vinicolor* genome assembly (Table S1). One of these putative alleles is located within a DOE-JGI gene model with predicted *ICMT* function (DOE-JGI protein ID# 750934), but both contigs containing putative *ICMT* alleles lack gene models or BLAST hits matching any other *B*-locus region genes. Initial BLAST searches of both *R. vinicolor* and *R. vesiculosus* identified many genes throughout both genomes with predicted *ICMT* activity, and it is not possible to determine if these putative *ICMT* alleles on small contigs in *R. vinicolor* represent alleles of *B*-locus region *ICMT* genes or other *ICMT* genes unlinked to the *B*-locus.

BLAST searches utilizing nucleotide sequences of *Rhizopogon B*-locus region genes as queries revealed likely alleles of these genes assembled on small contigs unlinked to the *B*-locus region in both *R. vinicolor* and *R. vesiculosus* (Table S1). The genes *RVSTE3.1* and *RVUP28*, which are found flanking one another in the *B*-locus regions of both *R. vinicolor* and *R. vesiculosus* (Figure 3 and File S7), produced BLAST hits adjacent to one another on small contigs separate from the *B*-locus of both *R. vinicolor* and *R. vesiculosus*. In *R. vesiculosus* these BLAST hits partially aligned to predicted gene models with the same relative transcriptional direction recorded for these genes on the primary *B*-locus contig. However, gene model predictions on this short contig (11.7 kbp) are truncated; producing no high quality BLAST matches to GenBank database proteins and only limited “noncytoplasmic” domain hits in Interproscan (https://www.ebi.ac.uk/interpro/). BLAST matches of *RVSTE3.1* and *RVUP28* alternate alleles in the genome assembly of *R. vinicolor* show similar concordance with expected transcriptional orientation, and the BLAST hit of *RVESTE3.1* overlaps an existing gene model (DOE-JGI protein ID# 699818) with a “STE3 mating receptor” activity predicted by Interproscan. We identified a putative pheromone precursor gene on the same short *R. vinicolor* contig using our Perl pheromone pattern matching script. Additionally, a short contig (1.3 kbp) was identified from the *R. vinicolor* assembly that contained a high quality BLAST match to the transmembrane domains region of the *RVSTE3.1* gene from *R. vesiculosus* (MAKER ID# Rves056.07660). This match falls within a truncated gene model with a PFAM predicted *STE* domain. It is possible that the regions on short contigs identified through BLAST searches for alternate alleles do represent true *B-locus* alleles of both *R. vinicolor* and *R. vesiculosus*. However, because of the truncated or otherwise low quality nature of the pheromone receptor gene models on these contigs, they were not included in phylogenetic or synteny analyses.

### Evolution of B-locus pheromone receptors and ICMT genes

Phylogenetic inference of the evolutionary relationships between pheromone receptor genes (Figure 5) indicate that both *Rhizopogon* species possess pheromone receptor genes belonging to two ancient *MAT* pheromone receptor clades (Figure 5: clades 1 and 2) shared among many Agaricomycetes (James *et al.* 2013; Kües 2015), as well as two later diverging subclades of clade 1 receptors (Figure 5) previously identified in *Pleurotus* species, *C. cinerea*, *Coprinopsis disseminatus*, *F. velutipes*, and *Lentinula edodes* (James *et al.* 2004b, 2006; Riquelme *et al.* 2005; Van Peer *et al.* 2011; Wu *et al.* 2013). Based upon phylogenetic topology, and placement of genes of known mating function, we have further divided previously defined subclades of clade 1 into four total clades, with previously defined subclade 1 here represented by clades 1A, 1B, and 1C, and previously defined subclade 2 represented by clade 1D (Figure 5). *R. vinicolor* and *R. vesiculosus* both possess pheromone receptor homologs in both clades 1 and 2, and clade 1 subclades. However, *R. vinicolor* possesses two clade 2 pheromone receptors and four clade 1 pheromone receptors, whereas *R. vesiculosus* possesses one and three, respectively. Clade 1B is the only clade that lacks genes of known mating function (Figure 5), and it is likely that pheromone receptor homologs in this clade all lack mating function. Clade 1C possesses many Agaricales homologs of known mating function, as well as an *R. vinicolor* homolog, but lacks homologs from most of the Boletales fungi, most notably all bipolar Boletales and *R. vesiculosus*.

The three bipolar Boletales species also lacked homologs within other subclades of pheromone receptor gene clade 1, with *C. puteana* lacking homologs in subclades 1A and 1C, *Su. granulatus* in 1C and 1D, and *Su. luteus* in all subclades inferred to possess mating-type function (subclades 1A, 1C, and 1D). *Su. luteus* possesses two paralogs in pheromone receptor gene clade 2, though the tetrapolar species *P. involutus* and *S. lacrymans* also possess multiple paralogs in this clade (Figure 5). The *B*-locus of *Su. brevipes* possesses near identical gene content and synteny with *Su. luteus* (Figure 3 and Figure 5), lending support to a bipolar breeding system in *Su. brevipes*. The tetrapolar Boletales possess homologs in all pheromone receptor clades inferred to have mating type function, as well as multiple paralogs within many pheromone receptor clades, with the exception of *S. lacrymans* and *Pisolithus* species, which lack homologs in clade 1C. Overall, the pheromone receptor phylogeny of Figure 5 supports the reduced number of pheromone receptor homologs in *R. vesiculosus*, *R. salebrosus*, *Su. brevipes*, and the bipolar species as a result of a loss of pheromone receptor genes in these species, rather than an expansion in *R. vinicolor* and the tetrapolar species. An exception to this trend is seen in *P. tinctorius*, which appears to have an expanded set of clade 1D homologs.

**Figure 6:**
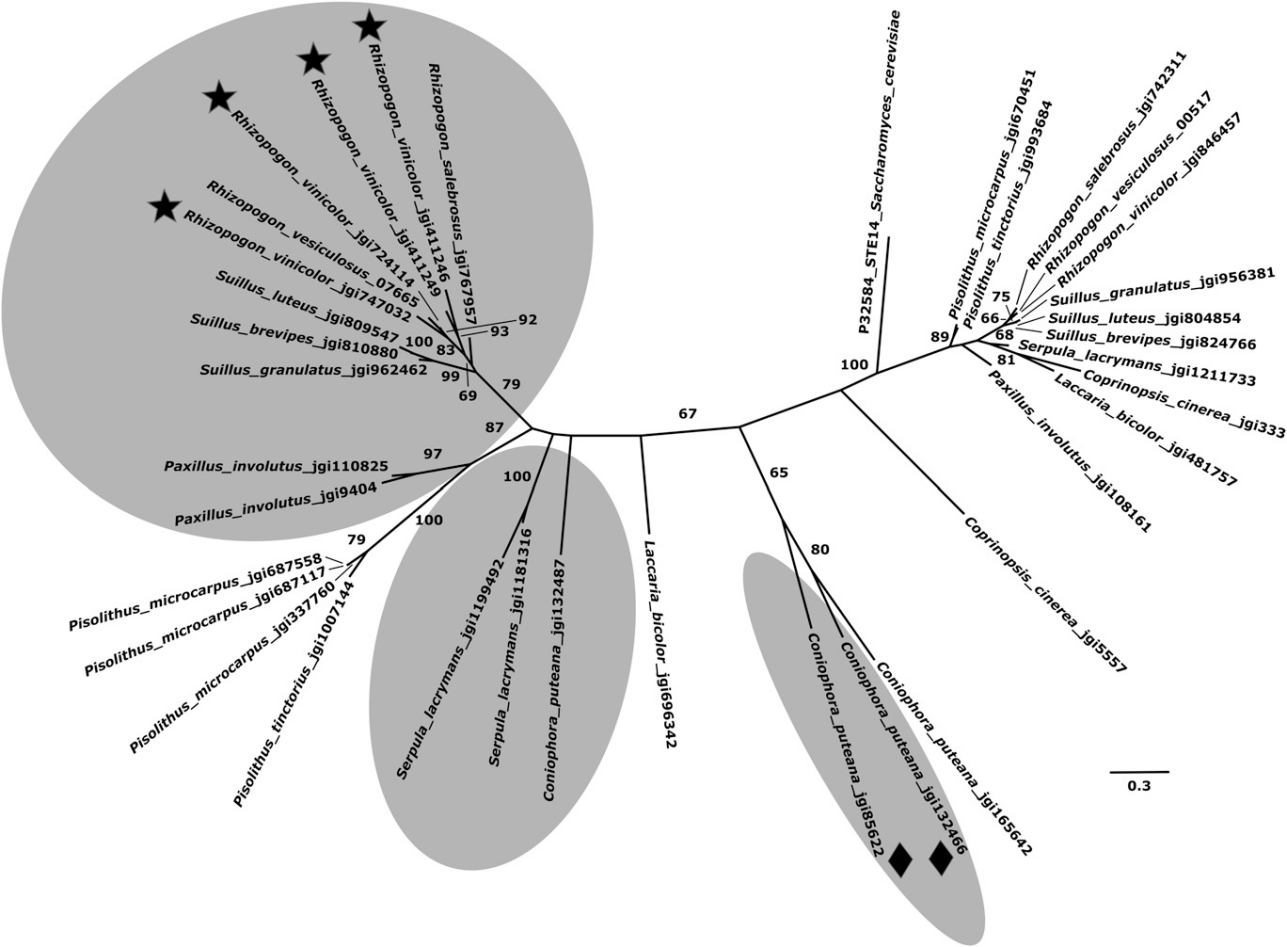
Maximum likelihood unrooted phylogram of *ICMT* genes inferred using RAxML with 500 bootstrap replicates and the PROTGAMMAGTR model of evolution. Bootstrap support values of ≥60% are shown for major branches. *ICMT* genes were identified as either having close proximity to the *B*-locus regions of the examined genomes or as sharing high BLAST sequence identity with the *B*-locus region *ICMT* genes of *R. vinicolor* or the *STE14* gene of *S. cerevisiae*. Genes identified in proximity to the *B*-locus regions are highlighted by gray circles. The two alleles of the *ICMT* genes that were present at a heterozygous break in *R. vinicolor B*-locus contigs are marked by stars. The two unique *ICMT* genes in *C. puteana* that are associated with a 130 kb translocation away from the main *B*-locus gene cluster are marked with diamonds. The *ICMT* genes denoted by stars and diamonds correspond to those *ICMT* genes marked with stars and diamonds in Figure 3.

We identified homologs of *ICMT* genes in close proximity to *B*-locus *P/R* genes in all Boletales genomes except *Pisolithus* species. While *ICMT* genes were not found near the *B*-locus of *Pisolithus* or Agaricales species, homologs of *ICMT* genes were identified from these genomes in other locations by performing BLAST searches with *R. vinicolor ICMT* genes as queries (Rhivi1 DOE-JGI protein ID#s: 747032, 724114, 411247, 411249). The best characterized *ICMT* protein known to function in mating-type recognition is the product of the pheromone maturation factor *STE14* from *Saccharomyces cerevisiae* (Caldwell *et al.* 1995), and additional *ICMT* genes were identified from all genomes by performing BLAST searches with a sequence of *STE14* as query (GenBank Accession #: P32584). The amino acid sequences of top scoring BLAST hits were aligned with *Rhizopogon ICMT* genes, and results of phylogenetic analysis are presented as an unrooted phylogram in Figure 6.

Phylogenetic analysis reveals that the pairs of *B*-locus region *ICMT* genes in *R. vinicolor* as well as those in tetrapolar Boletales reference genomes all cluster taxonomically. This indicates that duplication of *B*-locus region *ICMT* genes may have occurred within species. While *C. puteana* lacks a second *ICMT* gene in immediate proximity to *P/R* genes it does possesses additional *ICMT* duplicates 130 kb 3′ of the *B*-locus region—a feature unique to this species. The single *ICMT* gene of *R. vesiculosus* (MAKER ID: 7665) groups with a pair of the *R. vinicolor ICMT* homologs identified upon a single contig (DOE-JGI protein ID#s: 724114, 747032) (Figure 6). The pattern of *ICMT* duplication observed in these taxa is more consistent with a duplication of *ICMT* paralogs within taxa rather than a loss of *ICMT* genes in *R. vesiculosus*, *R. salebrosus*, and bipolar Boletales. All top BLAST hits to *STE14* were not in genetic proximity to the *B*-locus of their genomes. Figure 6 shows that *STE14* is grouped with 100% bootstrap support with all of its top BLAST hits from all genomes save for the hit identified from *C. puteana* (Conpu1 DOE-JGI protein ID#: 165642). Instead, the *STE14* BLAST hit in *C. puteana* groups with 95% bootstrap support, with other *C. puteana ICMT* genes located in the genomic translocation 130 kbp from the *B*-locus region. All *C. puteana ICMT* genes have greater phylogenetic similarity with Boletales *B*-locus region *ICMT* genes than with *STE14* (Figure 6).

Duplicate copies of the *B-locus* region *ICMT* gene in *R. vinicolor*, *S. lacrymans*, and *P. involutus*, and the lack of this duplicate in *R. vesiculosus*, *R. salebrosus*, and *C. puteana* may confer differential pheromone maturation pathways in these fungi. While gene duplication has been demonstrated as a potential source of genomic incompatibility resulting in reproductive isolation between sister taxa (Lynch and Force 2000), it is unlikely that speciation between *R. vinicolor* and *R. vesiculosus* was spurred by loss or gain of gene duplicates in the mating pheromone maturation pathway. Rather, a lack of paralogs could account for differential mating behavior leading to the observed pattern of reduced effective population size in *R. vesiculosus* (Dunham *et al.* 2013; Kretzer *et al.* 2005).

### Conclusion

In this study, we have examined the putative *MAT* loci from all available genomes of Boletales fungi with a known breeding system. Comparison of *B*-loci from heterothallic bipolar and tetrapolar Boletales species reveals a pattern of reduced gene content in bipolar genomes (Figure 4 and Figure 5), and we hypothesize that loss of genetic diversity and function at the *B*-locus region may be associated with the transition from tetrapolar to bipolar breeding systems in the Boletales. We have demonstrated differential gene content of *R. vinicolor* and *R. vesiculosus MAT B*-loci, which provides a degree of support for our hypothesis that differences in the *MAT* loci of *R. vinicolor* and *R. vesiculosus* may underlie observed differences in the population structure of these fungi. The *B*-locus of *R. vinicolor* shows the most similarity to that of the tetrapolar Boletales, with multiple *B*-locus region *ICMT* homologs (Figure 3 and Figure 6), pheromone receptor homologs in all phylogenetic clades with an additional paralog in clade 2 (Figure 5), and a greater number of pheromone precursor genes flanking pheromone receptor genes (Figure 3, Figure 4 and Table 4). *R. vesiculosus* possesses fewer homologs within pheromone receptor clades inferred to possess mating type function (Figure 5), fewer pheromone precursor genes (Figure 3, Figure 4 and Table 4), and only a single *B*-locus region *ICMT* gene (Figure 3 and Figure 6). Taken together, the features of the *R. vinicolor B*-locus are consistent with the features of tetrapolar Boletales, whereas *R. vesiculosus* possesses features, *i.e.*, reduced gene diversity, at its *B*-locus that are intermediate between bipolar and tetrapolar Boletales (Figure 4). Several partial pheromone receptor genes were found on short contigs separate from the primary *B*-locus contigs in the genomes of both *R. vinicolor* and *R. vesiculosus*. These partially assembled genes may represent highly divergent alleles of the *B*-locus pheromone receptors. providing support for a heterothallic breeding system in both *R. vinicolor* and *R. vesiculosus*.

It is hypothesized that bipolar breeding systems in Agaricomycetes have been derived multiple times from tetrapolar ancestors (Hibbett and Donoghue 2001; Nieuwenhuis *et al.* 2013). This transition has been observed in Basidiomycota through genetic linkage of the *A* and *B MAT* loci in *Ustilago hordei* (Ustilaginomycotina, Ustilaginaceae) (Bakkeren and Kronstad 1994), and through a loss of specificity or function of *B*-locus *P/R* genes in *C. disseminatus* (James *et al.* 2006). The observation of fewer pheromone receptor genes in bipolar Boletales genomes compared to tetrapolar Boletales genomes is consistent with a loss of function and subsequent loss of genetic diversity. The reduction in *B*-locus *P/R* gene content in *R. vesiculosus* compared to *R. vinicolor* is not as drastic as the difference between *R. vinicolor* and the bipolar species *Su. luteus*, *Su. granulatus*, and *C. puteana*. This might indicate that *R. vesiculosus* has lost some mating type pheromone receptor specificity in recent evolutionary history (*e.g.*, drift associated with reduced effective population size). It is also possible that we have selected a strain of *R. vesiculosus* for genome sequencing that simply possesses a deletion of *P/R* genes in both of its *B*-locus alleles or that our genome assembly was biased for only one *B*-locus allele that lacked *P/R* genes. The *P/R* genes of the Agaricomycetes *MAT B*-locus are highly variable between haplotypes, and lack, or truncation, of particular pheromone precursor genes has been observed for some *C. cinerea B*-locus haplotypes (Riquelme *et al.* 2005; Kües *et al.* 2015). However, it is more likely that *R. vesiculosus* truly possesses reductions in pheromone receptor genes, since these reductions are also observed in the bipolar species *Su. luteus*, *Su. granulatus*, and *C. puteana*.

Regardless of the breeding systems (heterothallic bipolar *vs.* heterothallic tetrapolar) operating in *R. vinicolor* and *R. vesiculosus*, further study of *MAT* alleles from additional strains is required to determine the mating systems [the degree of diploid selfing or outcrossing, sensu Billiard *et al.* (2012)] operating in these fungi. We hypothesize here that a change in the genomic structure of *MAT* loci governing the breeding systems of *R. vinicolor* and *R. vesiculosus* may affect their rates of outcrossing. However, an increased number of *MAT* alleles in a fungal population can also increase the degree of outcrossing within that population without any change in the breeding system (Nieuwenhuis *et al.* 2013). Thus, it is also likely that the differential population structure in natural populations of *R. vinicolor* and *R. vesiculosus* may be the product of higher allelic diversity at the *MAT* loci of *R. vinicolor*. The strain of *R. vinicolor* used in genome sequencing was drawn from a more readily outcrossing population than that of *R. vesiculosus* (Dunham *et al.* 2013; Kretzer *et al.* 2005), and this would likely function to maintain and increase the allelic diversity of *R. vinicolor MAT* loci in this population (Nieuwenhuis *et al.* 2013).

## Supplementary Material

Supplemental material is available online at www.g3journal.org/lookup/suppl/doi:10.1534/g3.117.039396/-/DC1.
