# Supplementary material for: Comparative Genomics of the Ectomycorrhizal Sister Species Rhizopogon vinicolor and Rhizopogon vesiculosus (Basidiomycota: Boletales) Reveals a Divergence of the Mating Type B Locus
Source: G3 (Bethesda). 2017 Apr 20;7(6):1775–89. doi: 10.1534/g3.117.039396 (PMC5473757; doi:10.1534/g3.117.039396)
Supplement: Supplementary file 1 [file 1775FileS1.doc]

**Comparative genomics of the ectomycorrhizal sister species *Rhizopogon* *vinicolor* and *Rhizopogon vesiculosus* (Basidiomycota: Boletales) reveals a divergence of the mating type *B* locus**

Alija Bajro Mujic^*1^, Alan Kuo^†^, Andrew Tritt^†^, Anna Lipzen^†^, Cindy Chen^†^, Jenifer Johnson^†^, Aditi Sharma^†^, Kerrie Barry^†^, Igor V. Grigoriev^†^, Joseph W. Spatafora^*^

* Oregon State University, Department of Botany and Plant Pathology. Corvallis, OR 97331

† United States Department of Energy, Joint Genome Institute. Walnut Creek, CA 95458

1. Current address: University of Florida, Department of Plant Pathology. Gainesville, FL 32611

**SUPPLEMENTARY METHODS**

**Culture conditions and tissue harvest:**

Cultures for DNA extraction were started by transfer of small sections (1 mm^3^) of *Rhizopogon* agar cultures into either sterile MMN broth (*R. vesiculosus*) (recipe of Kennedy et al. 2011 omitting agar) or MMN agar media (*R. vinicolor*) (Kennedy et al. 2011) topped with sterilized cellophane membrane (BioRad, Hercules, CA). Broth cultures were incubated at 25°C for 1 – 4 weeks with short daily applications of stirring to promote oxygenation and colony fragmentation. Once broth cultures reached 1 cm diameter fungal tissue was filtered from media by using a Buchner flask and Whatman filter paper (GE Healthcare Bio-Sciences, Pittsburg, PA). Agar residue from inoculation plugs was removed and remaining fungal tissue was blotted dry between two sheets of filter paper. Cellophane MMN cultures were incubated at 20°C for 1 – 2 weeks and tissue was harvested by directly peeling mycelium from the cellophane membrane. Harvested fungal tissues were flash frozen in liquid nitrogen, lyophilized, and stored at -80°C until the time of DNA extraction*.*

Production of tissue for RNA extraction was performed on four distinct culture media to promote a diverse population of RNA transcripts. Tissue production for RNA was performed for both species using solid agar media and cellophane membrane upon four media formulations: Potato Dextrose Agar (PDA) (BD Difco, Franklin Lakes, New Jersey), standard MMN (Kennedy et al. 2011) plus MES buffer, MMN media omitting MES buffer, and MMN media omitting B-vitamin supplement. Cultures were allowed to grow no longer than 7 days at 20°C from the time of initial growth after transfer. Cultures were then harvested by peeling mycelium from the cellophane membrane, flash frozen in liquid nitrogen, and stored at -80°C. Frozen tissue was stored for no more than 2 months before RNA extraction was performed.

**DNA and RNA extraction:**

For DNA extractions, lyophilized tissue was chilled with liquid nitrogen and pulverized either by using an autoclaved mortar and pestle or in a 1.5 ml microcentrifuge tube using autoclaved micropestles. Approximately 100 µl of finely ground fungal tissue was suspended in 2% CTAB buffer, incubated at 65°C for 60 minutes and then mixed 1:1 with 25:24:1 Phenol:Chloroform:Isoamyl alcohol (P:C:IAA) to extract DNA. Samples were centrifuged and the aqueous layer was separated and treated with 10 mg/ml RNAse A (multiple suppliers) for 1 hour at 35°C. RNAse treated samples were extracted a second time by mixing 1:1 with 24:1 C:IAA. DNA was precipitated from the supernatant by addition of 2 volumes 95% ethanol and 1/10 volume 3M sodium acetate.

RNA was extracted from flash frozen tissue following a combined TRIzol and RNeasy protocol. Frozen tissue was thawed directly in TRIzol reagent (Life Technologies, Grand Island, NY) in a lysing matrix D (MP biomedicals, Santa Ana, CA) bead beating tube. Samples were homogenized in a GenoGrinder 2000 device (Spex Sample Prep, Metuchen, NJ) using two rounds of shaking at 1750 RPM for 30 seconds with a cool down period of 1 minute between runs. Homogenized samples were combined with 0.2 volume chloroform and centrifuged. RNA was precipitated from supernatant using 0.5 volumes of isopropyl alcohol and RNA was resuspended in molecular biology grade water. The RNA solution was then cleaned using the RNeasy Mini Kit (Qiagen, Valencia, CA) following manufacturer protocols including the optional on column DNase treatment.

**Genome sequencing and assembly:**

Sequencing and assembly of the *R. vinicolor* genome was conducted at the United States Department of Energy Joint Genome Institute (DOE-JGI) (Walnut Creek, CA). For the genomic Illumina library, 100ng of DNA was sheared to 270bp using the Covaris LE220 (Covaris, Woburn, MA) and size selected using SPRI beads (Beckman Coulter, Indianapolis, IN). The fragments were treated with end-repair, *A*-tailing, and ligation of Illumina compatible adapters (IDT Inc., San Jose, CA) using the KAPA-Illumina library creation kit (KAPA Biosystems Inc., Wilmington, MA). An RNA-seq library was generated using the Illumina Truseq Stranded RNA LT kit. mRNA was purified from 1µg of total RNA using magnetic beads containing poly-T oligos. mRNA was fragmented and reverse transcribed using random hexamers and SSII (Life Technologies (Invitrogen), Grand Island, NY) followed by second strand synthesis. The fragmented cDNA was treated with end-pair, A-tailing, adapter ligation, and 10 cycles of PCR.

The prepared libraries were quantified using KAPA Biosystem’s next-generation sequencing library qPCR kit and run on a Roche LightCycler 480 real-time PCR instrument. The quantified libraries were then multiplexed into pools of 2 libraries each, and the pool was then prepared for sequencing on the Illumina HiSeq sequencing platform utilizing a TruSeq paired-end cluster kit, v3, and Illumina’s cBot instrument to generate a clustered flowcell for sequencing. Sequencing of the flowcell was performed on the Illumina HiSeq2000 sequencer using a TruSeq SBS sequencing kits, v3, following a 2x150 indexed run recipe.

Genome and transcriptome sequencing, assembly and annotation of *R. vesiculosus* were performed at Oregon State University (OSU). The Illumina DNA library used for genome sequencing was prepared using the NEBnext DNA Master Mix Kit for Illumina (New England Biolabs, Ipswich, MA) following the manufacturer’s protocol with size selection for a 420 bp library fragment size performed by agarose gel electrophoresis size selection. An RNASeq library was prepared for use in transcriptome sequencing using the Illumina TruSeq RNA sample preparation kit (Illumina Inc., San Diego, CA) following the manufacturer protocol.

**REFERENCES:**

Kennedy, P. G., L. M. Higgins, H. Rogers, and M. G. Weber. 2011. Colonization-Competition Tradeoffs as a Mechanism Driving Successional Dynamics in Ectomycorrhizal Fungal Communities. *PLoS ONE* 6(9): e25126. doi:10.1371/journal.pone.0025126.
