# Supplementary material for: Comparative Genomics of the Ectomycorrhizal Sister Species Rhizopogon vinicolor and Rhizopogon vesiculosus (Basidiomycota: Boletales) Reveals a Divergence of the Mating Type B Locus
Source: G3 (Bethesda). 2017 Apr 20;7(6):1775–89. doi: 10.1534/g3.117.039396 (PMC5473757; doi:10.1534/g3.117.039396)
Supplement: Supplementary file 8 [file 1775FileS8.doc]

File S1. Supplementary Methods. This file contains detailed descriptions of nucleic acid extraction, Illumina library construction, and Illumina library sequencing.

File S2. pheromone_seeker.pl. This Perl script identifies open reading frames with terminal -CAAX motifs in DNA sequences supplied by the user.

File S3. protein_coordinates.pl. This Perl script returns genome coordinates for a list of gene models or all gene models from a given genome contig.

File S4. protein_select_by_contig.pl. This file is a Perl script which returns amino acid sequence data for all proteins encoded by gene models on a given contig or from a list of gene models supplied by the user.

File S5. select_by_contig_region.pl. This file is a Perl script which returns nucleotide sequence data from genome coordinates specified by the user.

File S6. SNP_density_calc.pl. This file is a Perl script which calculates the effect of SNPs upon the predicted amino acid sequences of proteins encoded by given gene models. The script takes as input .gff3 format gene model predictions and SNP calls produced by the software VARSCAN2.

File S7. Gene model coordinates. This file is an excel spreadsheet listing coordinates of all predicted gene models in the *A* and *B* locus regions of all genomes examined in this study.

Table S1. Alternate *B*-locus alleles. This table contains genome coordinates for alternate alleles of *B-*locus region genes identified in the genomes of *Rhizopogon vinicolor* and *Rhizopogon vesiculosus*. The file contains coordinates of pheromone receptor, pheromone precursor, and *B-*locus region genes identified from small contigs unlinked to the main *B-*locus contigs identified in these genomes. Table S1 is in excel spreadsheet format.
